# Enhanced Early Neuronal Processing of Food Pictures in Anorexia Nervosa: A Magnetoencephalography Study

**DOI:** 10.1155/2016/1795901

**Published:** 2016-07-25

**Authors:** Lauren R. Godier, Jessica C. Scaife, Sven Braeutigam, Rebecca J. Park

**Affiliations:** ^1^Oxford Brain-Body Research into Eating Disorders, Department of Psychiatry, University of Oxford, Oxford OX3 7JX, UK; ^2^Oxford Centre for Human Brain Activity, Department of Psychiatry, University of Oxford, Oxford OX3 7JX, UK

## Abstract

Neuroimaging studies in Anorexia Nervosa (AN) have shown increased activation in reward and cognitive control regions in response to food, and a behavioral attentional bias (AB) towards food stimuli is reported. This study aimed to further investigate the neural processing of food using magnetoencephalography (MEG). Participants were 13 females with restricting-type AN, 14 females recovered from restricting-type AN, and 15 female healthy controls. MEG data was acquired whilst participants viewed high- and low-calorie food pictures. Attention was assessed with a reaction time task and eye tracking. Time-series analysis suggested increased neural activity in response to both calorie conditions in the AN groups, consistent with an early AB. Increased activity was observed at 150 ms in the current AN group. Neuronal activity at this latency was at normal level in the recovered group; however, this group exhibited enhanced activity at 320 ms after stimulus. Consistent with previous studies, analysis in source space and behavioral data suggested enhanced attention and cognitive control processes in response to food stimuli in AN. This may enable avoidance of salient food stimuli and maintenance of dietary restraint in AN. A later latency of increased activity in the recovered group may reflect a reversal of this avoidance, with source space and behavioral data indicating increased visual and cognitive processing of food stimuli.

## 1. Introduction

Anorexia Nervosa (AN) is a severely debilitating psychiatric disorder characterized by the relentless pursuit of thinness, leading to severe emaciation. It is associated with low rates of recovery [1] and has the highest mortality rate of any psychiatric disorder [2]. AN is extremely challenging to treat [3], and a lack of evidence-based treatments [4] has increased interest in investigating the neurobiological mechanisms underlying AN [5, 6].

Neuroimaging evidence suggests that food stimuli are aberrantly processed in AN. Increases in activity in the ventral limbic stream of emotional processing have been reported in response to food images in AN compared to controls [7–10]. This network is involved in the identification of emotionally salient stimuli and affective states [11, 12], suggesting food stimuli have emotional significance to individuals with AN. Furthermore, our group has previously shown an increased neural response to both rewarding and aversive food taste stimuli even after recovery from AN, suggesting increased salience attribution is given to food stimuli in these individuals [13].

Motivationally and emotionally relevant stimuli are suggested to lead to greater attentional processing [14]. Indeed, an attentional bias for food stimuli is reported in AN, using behavioral tasks such as the Emotional Stroop Task, Visual Dot Probe tasks, and Startle Reflex paradigms [15, 16]. However, evidence from eye tracking indicates that, despite initial attentional engagement, individuals with AN allocate less overall attention to food pictures compared to healthy controls, indicating an initial attentional bias may be followed by avoidance in these individuals [17]. These temporally distinct attentional processes in AN cannot be fully captured by functional Magnetic Resonance Imaging (fMRI), which has low temporal resolution and requires further investigation.

Electrophysiological measures of neural processing, such as electroencephalography (EEG) and magnetoencephalography (MEG), provide a real-time measure of neural activity and as such can map precisely the temporal dynamics of attentional processes. Event-Related Potentials (ERP) at different latencies are suggested to reflect different aspects of the information processing stream. For example, ERP measures of attention found at an early latency (100–300 ms after stimulus; e.g., N100/P100) are suggested to reflect early sensory processing and attentional orienting/selection processes [18]. ERPs in the midrange (200–300 ms after stimulus; e.g., early posterior negativity, EPN/N200/P200) are associated with emotional arousal and selective processing of emotional stimuli [19, 20]. Lastly, ERPs in the later range (300 ms and above after stimulus; e.g., late positive potential, LPP/P300) reflect the incentive salience or motivated attention towards a stimulus [18, 21]. Despite neuroimaging and behavioral evidence of the increased salience of food stimuli in AN, there are a few EEG studies investigating the neural processing of food stimuli in AN, with inconsistent results [22–24], and no published MEG studies to date. With this in mind, the current study aimed to extend the investigation of the temporal dynamics of the neural processing of food stimuli in individuals with AN using MEG, in conjunction with a reaction time task and eye tracking.

There is also evidence to suggest that individuals with AN process high- and low-calorie food stimuli differently, which may underpin the underconsumption of high-calorie food and overconsumption of low-calorie food that characterizes AN. In a behavioral study, our group found that individuals with AN consciously desire and implicitly “want” low-calorie foods more than high-calorie foods. This is consistent with differences in the neural response to high- versus low-calorie foods in AN in reward and emotion processing regions in AN [25, 26]. Moreover, in a recent fMRI study, we found differential hyperactivation of the lateral frontal pole in AN versus healthy controls in response to high-calorie compared to low-calorie food pictures (unpublished data). The frontal pole serves as a supervisory attentional control centre [27, 28]. As such, hyperactivation during the high-calorie food pictures may reflect that, even in their malnourished state, individuals with AN feel the need to resist the biological drive to eat when energy-dense foods threaten their extreme dietary control [29].

In light of these results, we chose to investigate the neural response to both high- and low-calorie food pictures in AN. Participants included both individuals currently ill and recovered from AN, as this enables investigation of both state and trait differences in the processing of food stimuli in AN. It was hypothesized that high- and low-calories food pictures would be experienced as highly salient for both individuals currently ill and recovered from AN reflected by increased neural processing after stimulus for both high- and low-calorie food pictures in comparison to healthy controls. This was hypothesized to be further reflected by group differences in the behavioral reaction time and eye-tracking measures. We also predicted a difference would be seen in the neural response to high-calorie compared to low-calorie foods in the AN groups, reflecting previous fMRI results and behavioral differences in the implicit and explicit motivation for high- and low-calorie foods in AN.

## 2. Methods

### 2.1. Participants

Forty-two participants were recruited for 3 experimental groups; current AN (AN-C) (*n* = 13), recovered from AN (AN-R) (*n* = 14), and healthy controls (HC) (*n* = 15). General exclusion criteria included age <18 or >60, insufficient English language comprehension, male sex, left-handedness, and any contraindication to having a Magnetic Resonance Imaging (MRI) scan (head injury, neurological or any other severe medical illness, pregnancy, and metal implants of any kind). Ethical permission for this study was obtained from the South Central-Oxford A Research Ethics Committee.

#### 2.1.1. AN-C Group

Inclusion criteria for the AN-C group included a current diagnosis of restrictive AN according to the Diagnostic and Statistical Manual of Mental Disorders, 4th edition (DSM-IV). Participants with a history or current diagnosis of binge/purge type AN or Bulimia Nervosa (BN) were excluded. Four participants were on both antidepressant (Selective Serotonin Reuptake Inhibitors [SSRI] or Serotonin Noradrenaline Reuptake Inhibitors [SNRI]) and atypical antipsychotic medication, and two were taking just antidepressant (SSRI) medication. Seven participants currently met the criteria for a mood disorder, 7 met the criteria for current Generalized Anxiety Disorder (GAD), and two met the criteria for current Obsessive Compulsive Disorder (OCD). Three participants reported previous but not current episodes of depression and 1 participant reported a previous episode of Posttraumatic Stress Disorder (PTSD).

#### 2.1.2. AN-R Group

Inclusion criteria for the AN-R group included a past diagnosis of restrictive AN according to the DSM-IV. Participants with a history of binge/purge type AN or BN were excluded. In addition, participants must have maintained a body mass index (BMI) between 18.5 and 25 for the previous 12 months (assessed by self-report) and score within 1 SD of the Eating Disorder Examination (EDE) and Eating Disorder Examination-Questionnaire (EDE-Q) global mean scores for young women and not met current criteria for any DSM-IV disorder as assessed by the Structured Clinical Interview for the DSM-IV (SCID). Whilst none of the AN-R participants currently met criteria for depression, 12 had previously met the criteria for depression, and 5 were currently taking antidepressant medication. In addition, 3 participants had previously met the criteria for GAD and 1 for OCD.

#### 2.1.3. HC Group

Inclusion criteria for the HC group included a current BMI between 18.5 and 25, no first-degree relative with a current or past ED diagnosis, no current or past diagnosis of a DSM-IV disorder, and scores within 1 SD of the EDE and EDE-Q mean scores for young women.

### 2.2. Procedures

Participants were initially screened by email to ensure they met the criteria for the study and then invited to a screening session at the Department of Psychiatry, University of Oxford. After informed consent was taken, participants were screened for current DSM-IV Axis-I disorders and given a battery of questionnaires to complete (described below).

Participants subsequently attended two more sessions in order to complete the MEG scan at the Oxford Centre for Human Brain Activity (OHBA) and the Oxford Centre for Magnetic Resonance Imaging (OCMR) for a Magnetic Resonance Imaging (MRI) structural scan. Participants were asked not to eat for 8 hours prior to the MEG scan. Previous studies have suggested that food deprivation may increase the activation of the appetitive motivational system [30]. As such, we predict it is in the fasted state that psychopathological processes will be most naturalistic for those with current AN. Subsequent to the MEG scan, participants completed subjective ratings of a number of food pictures they had seen in the scanner.

### 2.3. Measures

The SCID [31] was used to screen for Axis-I disorders. Eating disorder (ED) symptoms were measured using the global mean scores on the EDE [32], EDE-Q [33], and Self-Starvation Scale (SS) [34]. The Clinical Impairment Assessment (CIA) [35] was used to assess the severity of ED-related psychosocial impairment. Depression symptoms were measured using the Beck Depression Inventory (BDI-II) [36]. Anxiety symptoms were measured using the State Trait Anxiety Scale (STAI) [37]. Impulsivity was measured using the Barratt Impulsiveness Scale (BIS) [38]. Compulsivity was measured using the Obsessive Compulsive Inventory Revised (OCI-r) [39] and the Yale-Brown-Cornell Eating Disorder Scale Self-Report Questionnaire (YBC-EDs-SRQ) [40]. Participants also completed the Sensitivity to Punishment and Reward Questionnaire (SRSPQ) [41]. Verbal IQ was assessed using the National Reading Test (NART) [42]. Height and weight were taken to calculate BMI, and participants in the AN groups provided information regarding duration of illness, lowest BMI, treatments received, age of onset, and duration of recovery if applicable. To assess subjective states before the MEG scan, participants completed a series of 100 mm visual analogue scales (VAS) assessing in the moment mood (happiness, sadness, and anxiety), appetite related variables (hunger, thirst, fullness, and desire to eat), and ED-related variables (current body image, fear of gaining weight).

### 2.4. MEG Scan

MEG data was acquired using an Elekta VectorView*™* MEG system at OHBA. The system provides a total of 306 channels; however, only data from the 204 gradiometers were considered here. The gradiometers are most sensitive to nearby (cortical) sources, where the local root-mean-square (RMS) value calculated over the two channels at each detector site and the global RMS value calculated over all channels are measures of the local and global brain activity, respectively. Accordingly, global RMS curve showed neuronal activity as a function of time, whereas local RMS maps illustrate the distribution of neuronal activity over the head at a given point in time.

On arrival, participants were screened for any contraindications to MEG and completed the VAS measures. They were then asked to sit in the scanner briefly to check for any artefacts in the MEG signal. Prior to the scan, two electrodes were placed next to the left eye to record electrooculogram (EOG) data, two electrodes were placed on the wrist to record electrocardiogram (ECG) data, and a grounding coil was placed in the centre of the forehead. Standard Elekta-Neuromag HPI (Head Position Indicator) was used for coregistration with MRI and head tracking during scan. Binocular eye-tracking data was recorded by means of an EyeLink 1000 (SR Research) device, which was set up and calibrated once the participants were in the scanner.

### 2.5. Food Pictures Task

Whilst in the MEG scanner, participants completed a simple food pictures task. Stimuli were 40 high-resolution (1034 × 768), standardized digital color photographs of foods divided equally into high-calorie and low-calorie categories. These images were provided by L. Charbonnier of the Image Sciences Institute, UMC Utrecht, and created as part of the Full4Health project (http://www.full4health.eu/), funded by the European Union Seventh Framework Program (FP7/2007–2013) under Grant agreement no. 266408. See supplementary materials (in Supplementary Material available online at http://dx.doi.org/10.1155/2016/1795901) for an example of the high- and low-calorie food pictures viewed by participants. The food pictures were presented via the Presentation software package and projected onto a screen via a Panasonic DLP based projector PT-D7700E (viewing distance 1.2 m, screen size 54.5 × 43 cm, resolution 1280 × 1024, and frequency 60 Hz). The order of the pictures was randomized and repeated three times. Each picture was on screen for 4000 ms, with an interstimulus interval of 1300 ± 500 ms, during which a fixation cross was shown centrally. During each food picture, a small black square appeared centrally between 500 and 1500 ms after stimulus onset. Participants were required to respond by pressing a button as soon as they saw the black square, and these reaction times were recorded via a fORP 932 interface box system. The task took approximately 10 minutes to complete.

Previous research from our group suggests that, despite no significant difference in explicit “liking” responses for high- and low-calorie foods, individuals with AN show increased “wanting” (incentive salience) for low-calorie foods compared to high-calorie foods, with the inverse pattern to that seen in controls [43]. For that reason, participants in this study were instructed to inwardly think about how much they wanted to eat each of the foods shown at that moment in time, although it is acknowledged that it was not possible to ensure that participants followed this instruction.

### 2.6. Subjective Rating of Food Pictures

To investigate group differences in subjective ratings of high- and low-calorie food pictures, participants subsequently rated 12 of the food pictures seen in the scanner (6 high-calorie and 6 low-calorie) on a 10-point VAS scale on the following two questions:

1. How pleasant would it be to eat this food right now? (0: not pleasant, 10: very pleasant).
2. How much do you want to eat this food right now? (0: not at all, 10: very much so).

### 2.7. MRI Structural Scan

An MRI structural scan was performed at the OCMR using a 3-Tesla Siemens Trio scanner with a 32-channel head coil. High-resolution T1-weighted images were acquired for subject alignment, using an MPRAGE sequence (174 × 192 × 192 matrix, voxel resolution 1 mm^3^, TR = 2040 ms, TE = 4.7 ms, inversion time, and TI = 900 ms).

### 2.8. Data Analysis

Reaction time, subjective ratings, VAS scales, and questionnaire data were analyzed in the IBM Statistical Package for Social Sciences (SPSS) using between-subject analysis of variance (ANOVA). Reaction time data from three participants in the AN-C group was missing due to equipment failure during the experiment. The threshold for statistical significance was set at *p* < 0.05 for all analyses.

#### 2.8.1. Eye-Tracking Analysis

Right eye data (*x* and *y* deflection, pupil size) were analyzed using a measure of the variation in the raw data (span) calculated as the difference between the maximum and minimum of values (position measure 11.2.3) [44]. Eye-tracking data from one participant in the AN-R group had to be excluded due to high levels of eye-blink interference in the data. Correlations between eye-tracking data and MEG data were carried out using Spearman's Rho in SPSS. The threshold for statistical significance was set at *p* < 0.05 for all analyses. Results are reported as* X*-span,* Y*-span, and* P*-Span to reflect* x* and* y* deflection and pupil size, respectively.

#### 2.8.2. MEG Analysis

*(1) Acquisition.* Measurements were performed using a Neuromag-306 VectorView system. The data were sampled at 1000 Hz (0.03–330 Hz antialias filter; external artefact correction using Maxfilter*™*) and corrected for variations in head position before using MaxMove*™*. Physiological artefacts were identified from the recorded EOG and ECG and removed from the raw data using a projection method based on principal components (in-house software, algorithm similar to the spatial confound, and sensor data correction routines provided by SPM8; see also [45]). Remaining artefacts were manually excluded from the raw data (<2% of data). For averaging and subsequent analysis, data epochs from −0.3 to 2.5 s relative to stimulus onset were calculated separately for high- and low-calorie trials, band-pass filtered (1–30 Hz), and normalized to unit baseline (−0.1 to 0 s).

*(2) Time-Series Analysis*. Statistical differences in event-related fields (ERFs) between the conditions of interest across participants were analyzed employing a time-dependent measure *P*(*t*), the details of which have been fully specified elsewhere [46]:

(1) $$\begin{array}{c} P\left(\;t\;\right)=\text{probability}\left(\;\chi^{2}\;\right),\;\chi^{2}=-2\overset{N}{\underset{i=1}{\sum }}\ln\left[f_{i}\left(t\right)\right]. \end{array}$$

*N* denotes the number of channels. For each channel, *f*_*i*_(*t*) denotes a nonparametric statistical test applied to evoked fields of interest. For each significant interval (*P*(*t*) < 0.01), the spatial distribution of the significant difference was given by *f*_*i*_. Correspondence between the spatial distribution of the significant difference and the spatial distribution of ERFs (using grand-average power maps = root-mean-square of the two gradiometers in a pair) was explored. Two different nonparametric tests (*f*) were used in the formula above. A nonparametric ANOVA (3 groups × 2 conditions) served as the main statistical test followed by Mann-Whitney *U* post hoc tests for between group analyses.

The ANOVA was based on standard algorithms applied to rank-transformed data. This is a well-known and validated technique commonly used to fit nonnormal data into framework of conventional statistical theory [47]. Note that *P*(*t*) is a measure of the difference between brain signals (magnetic fields) which are related to but not necessarily the same as the difference between neuronal activities (here, squared readings). For simplification and robustness, significance (peaks of *P*) was further considered only if it was concomitant with a change in power (i.e., RMS signal; also tested with nonparametric statistics). Thus, the data presented here are indicative of both a change in dynamics (signal) and strengths of activity. As a consequence, some significant peaks (measure *P*) were not further analyzed because the simultaneous change in activity was not clear. Furthermore, latencies of above 500 ms were excluded due to potential interference from participants' button press response to the black square, which appeared 500–1500 ms after stimulus.

*(3) Source Analysis.* Source estimates were obtained for all subjects using S-Loretta inversion based on individual structural images. The head volume was approximated with overlapping spheres fitted to the local curvature. Voxel-based *t*-tests were used for statistical comparisons across groups. All source calculations were performed with Brainstorm 3.2 [48], which is documented and freely available for download online under the GNU general public license. The threshold for statistical significance was set at *p* < 0.05. The source analysis has certain limitations. Notably, it was not possible to establish significance after correcting for multiple comparisons (over voxels) due to strong intersubject variability in these data, which is most likely explained by the complex nature of stimuli used. Also, a lack of statistical power might account here for relatively weak source results. Note that the time-series analysis utilizes only data measured without interpolation typically required for source analysis (where thousands of voxel values are derived from hundreds of channel readings). Therefore, results in source space were restricted to times at which significance was established in signal space.

#### 2.8.3. Correlations with MEG Time-Series Data

Correlations were investigated between MEG time-series data and reaction times, subjective ratings, eye-tracking data, and questionnaire measures of ED symptomology (EDE, EDE-Q, CIA, and SS) using Spearman's Rho in SPSS. Due to neuroimaging evidence of increased activity in regions associated with cognitive control and response inhibition, correlations were also carried out with questionnaire measures of impulsivity and compulsivity (BIS, OCI-r, and YBC-EDS-SRQ). MEG time-series data for each participant was extracted by averaging activity over the peak intervals at *p* = 0.01 for time points of interest. Due to the focus on group differences in the current study, correlations were carried out separately for each experimental group.

## 3. Results

Participant demographics and psychological characteristics can be seen in Table 1.

### 3.1. VAS Scales

VAS scale scores for each experimental group can be seen in the supplementary materials. There were significant group differences in happiness (*F*[2,41] = 3.679, = 0.034), hunger (*F*[2,41] = 6.465, *p* = 0.004), desire to eat (*F*[2,41] = 6.503, *p* = 0.004), and fear of fatness (*F*[2,41] = 16.03, *p* < 0.001). Bonferroni corrected post hoc analysis indicated significantly higher happiness, hunger, and desire to eat in the HC compared to AN-C group (*p* < 0.05). Fear of fatness followed a pattern of AN-C > AN-R > HC (*p* < 0.05).

### 3.2. Behavioral Results

#### 3.2.1. Subjective Food Picture Ratings

Subjective ratings of pleasantness and wanting for high- and low-calorie food pictures stratified by experimental groups can be seen in the supplementary materials. There were a significant Calorie *∗* Group interaction in pleasantness ratings (*F*[2,39] = 5.916, *p* = 0.006) and a significant main effect of group (*F*[2,39] = 10.44, *p* > 0.001). This was driven by higher pleasantness ratings for both high- and low-calorie food pictures in the HC group compared to the AN-C and AN-R group (*p* < 0.05). There was also a significant main effect of calorie condition, indicating that all groups rated the high-calorie pictures as more pleasant than the low-calorie pictures (*F*[1,39] = 39.239, *p* < 0.001). There was no significant Group*∗*Calorie interaction (*F*[2,39] = 2.630, *p* = 0.085) or main effect of group (*F*[2,39] = 1.161, *p* = 0.364) in wanting ratings for food pictures. This suggests there was no difference between groups on ratings of wanting for high- or low-calorie food pictures. There was however a main effect of calorie category, with all groups indicating higher wanting for high-calorie compared to low-calorie food pictures (*F*[1,39] = 14.824, *p* < 0.001).

#### 3.2.2. Reaction Times

Reaction times to high- and low-calorie food pictures across experimental groups can be seen in the supplementary materials. There was no significant group effect (*F*[5,72] = 2.087, *p* = 0.131), calorie condition effect (*F*[1,72] = 1.669, *p* = 0.201), or Group*∗*Calorie condition interaction (*F*[2,72] = .128, *p* = 0.880) in reaction times to the food pictures.

#### 3.2.3. Eye Tracking

**Figure 1:**
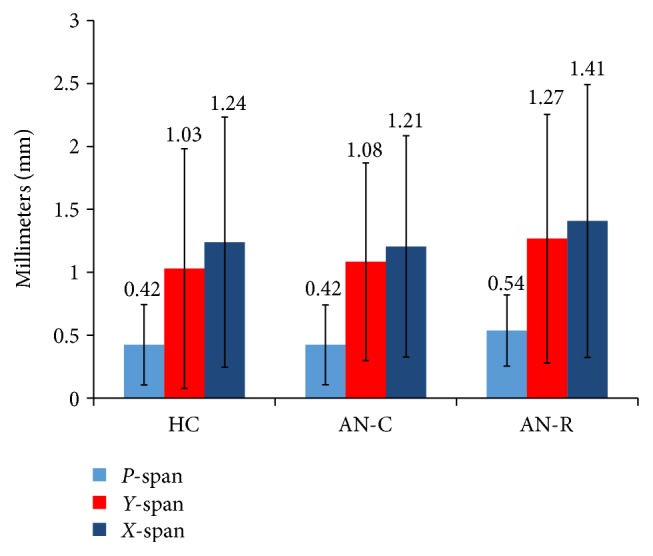
The main effect of group on *P*-span, *Y*-span, and *X*-span. *P*-span scores reflect pupil dilation (in mm) after stimulus onset. *Y*-span and *X*-span scores reflect movement horizontally and vertically (mm) from the central point of the picture. A significant main effect of group was found for all three measures (*p* < 0.05). Post hoc Bonferroni corrected *t*-tests indicated this was driven by increased scores on all three measures in the AN-R compared to the AN-C and HC groups (*p* < 0.05). AN-C: acute Anorexia Nervosa group, AN-R: recovered Anorexia Nervosa group, and HC: healthy control group.

*X*-span, *Y*-span, and *P*-span scores for each group can be seen in the supplementary materials. Significant group differences were found in the exploration of food pictures in general (both high and low conditions versus baseline). Group differences are shown in Figure 1 for the three measures of eye movement: *X*-span (*F*[2,2130] = 8.487, *p* < 0.001), *Y*-span (*F*[2,2130] = 13.143, *p* < 0.001), and *P*-span (*F*[2,2130] = 31.185, *p* < 0.001). *X*- and *Y*-span denote movement of the eye from the central position of the black star on the *x*- and *y*-axis. *P*-span denotes change in pupil size. Bonferroni corrected post hoc analyses suggested significantly higher scores in the AN-R group compared to both the AN-C and HC groups in *X*-span, *Y*-span, and *P*-span (*p* < 0.05). There was also a main effect of calorie condition on *X*-span (*F*[1,2130] = 20.472, *p* < 0.001), *Y*-span (*F*[1,2130] = 13.791, *p* < 0.001), and *P*-span (*F*[1,2130] = 3.847, *p* = 0.05), suggesting all participants explored high-calorie food pictures more than low calorie food pictures. There were no significant Group*∗*Calorie condition interactions (*X*-span: *F*[2,2130] = 0.270, *p* = 0.763; *Y*-span: *F*[2,2130] = 0.458, *p* = 0.663; *P*-span: *F*[2,2130] = 0.555, *p* = 0.574).

### 3.3. MEG Results

#### 3.3.1. Time-Series Analysis

**Figure 2:**
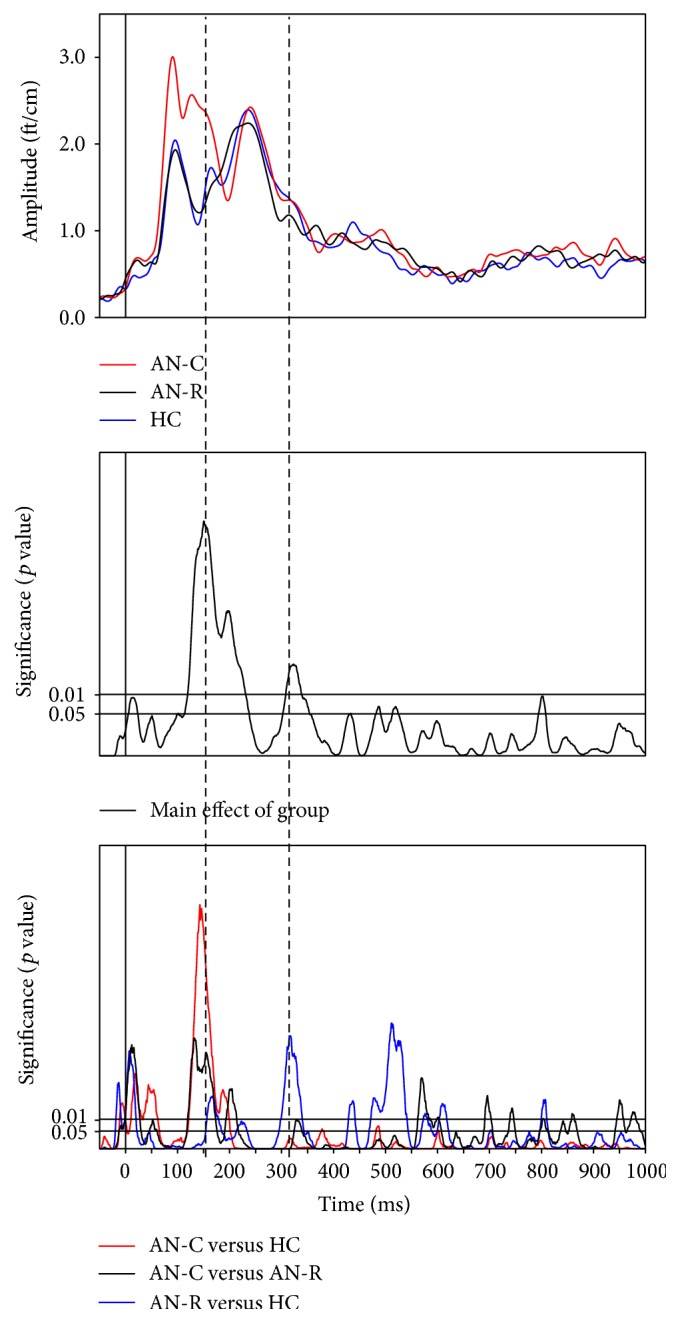
Group differences in neural activity. The top panel shows the mean amplitude (ft/cm) of neural activity after stimulus (collapsed across food categories) across the experimental groups. The middle panel illustrates the significance levels over time of the ANOVA main effect of group. The bottom panel illustrates the significance levels of post hoc Mann-Whitney *U* test analyses of group differences in mean amplitude of neural activity (collapsed across food categories) shown in the top panel. Note: - - - - - - denotes the two peaks of interest: 150 and 320 milliseconds after stimulus, in which group comparisons were significant to the *p* < 0.01 level (see Methods for justification of peak selection). AN-C: acute Anorexia Nervosa group, AN-R: recovered Anorexia Nervosa group, and HC: healthy control group.

**Figure 3:**
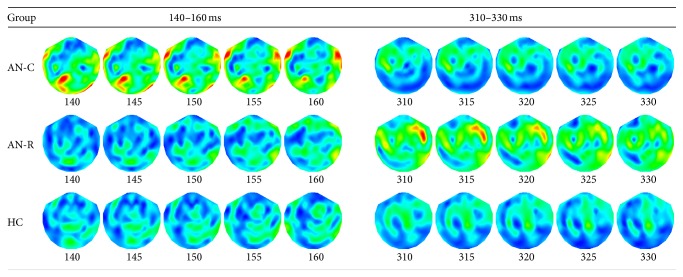
Mean amplitude of neural activity (ft/cm) for the two peak times of interest shown in sensor space (gradiometers) for each experimental group. Neural activity is shown with 10 milliseconds either side of the 150 and 320 milliseconds peaks after stimulus (all food pictures versus baseline). The scale used was 0–5 ft/cm with the exception of the 310–330 milliseconds' time period in the AN-R group, for which a scale of 0–3 ft/cm was used for visualisation purposes. AN-C: acute Anorexia Nervosa group, AN-R: recovered Anorexia Nervosa group, and HC: healthy control group.

Time-series analysis of the MEG data indicated a significant main effect of group in response to all food pictures (high- and low-calorie conditions) compared to baseline (see Figure 2, middle panel). Post hoc analyses (Mann-Whitney *U* tests) identified two significant time points of interest at which group differences reached a significance of *p* < 0.01: 150 ms and 320 ms. A significant increase in neuronal activity at 150 ms over posterior regions of the brain was observed in the AN-C group, compared to both the AN-R and HC groups. A significant increase in neuronal activity at 320 ms was also observed in the AN-R group, compared to both the AN-C and HC groups, over occipital regions of the brain. No significant main effect of calorie condition or Group*∗*Calorie condition interaction was found. Figure 2 shows the amplitude of neural activity over time collapsed across calorie conditions in each experimental group (top panel), the significance of the main effect of group over time (middle panel), and the significance of the Mann-Whitney *U* test group comparisons over time (bottom panel).  Figure 3 shows the amplitude of neural activity across the experimental groups in signal space at 140–160 ms and 310–330 ms after stimulus (collapsed across calorie conditions).

#### 3.3.2. Source Analysis

As explained in Section 2, the source analysis is somewhat limited and the results should be treated with care. We feel, however, that the clinical data here is worth presenting given the novelty of approach.

**Figure 4:**
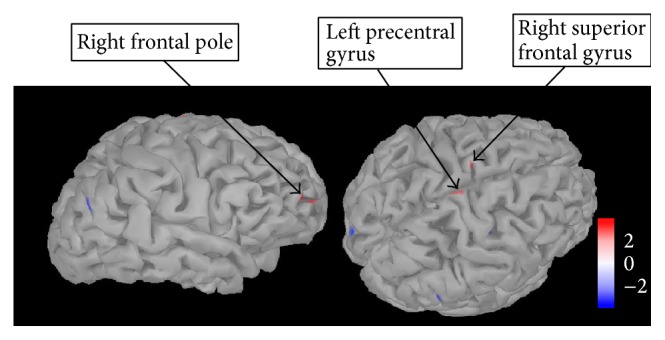
Regions showing significantly higher activation in the AN-C compared to HC group at 150 ms after stimulus. The figure shows the regions showing significantly higher activation in a *t*-test comparing the AN-C and HC group (shown in red) at 150 ms after stimulus (all food pictures versus baseline). Regions were identified by eye and are uncorrected for multiple comparisons (*p* = 0.005). Scale shown represents the *t*-value. AN-C: current Anorexia Nervosa group, HC: healthy control group.

*(1) Source Analysis of Neural Response at 150 ms*. As a significant difference in neural activity at this time point was identified between the AN-C and HC group and the AN-C and AN-R group, differences in source space were investigated in Brainstorm using between-group *t*-tests. As the AN-C group showed increased neural activity compared to the HC and AN-R group, the focus of the source space analysis was also limited to regions of increased activation.  Figure 4 shows the regions of increased activation in the AN-C compared to the HC group at 150 ms after stimulus (shown in red; *p* = 0.005). These regions were identified as the left precentral gyrus (Brodmann area 10), right frontopolar cortex (Brodmann area 10), and right superior frontal gyrus (Brodmann area 6).

**Figure 5:**
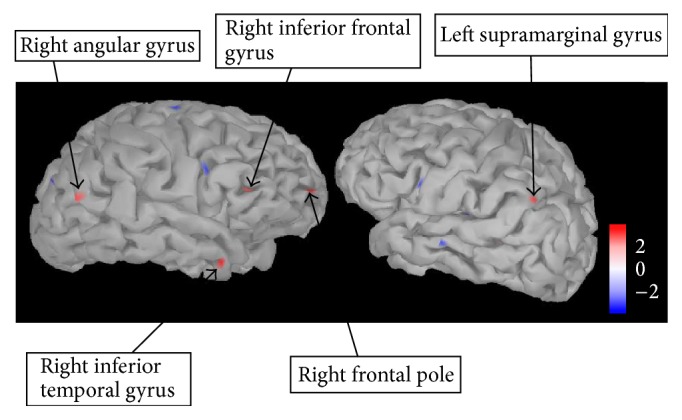
Regions showing significantly higher activation in the AN-C compared to AN-R group 150 ms after stimulus. Regions showing significantly higher activation in a *t*-test comparing the AN-C and AN-R group (shown in red) at 150 ms after stimulus (all food pictures versus baseline). Regions were identified by eye and are uncorrected for multiple comparisons (*p* = 0.005). Scale shown represents the *t*-value. AN-C: current Anorexia Nervosa group, AN-R: recovered Anorexia Nervosa group.

Figure 5 shows the regions with increased activation in the AN-C compared to the AN-R group at 150 ms after stimulus (shown in red; uncorrected, *p* = 0.005). These regions were identified as the bilateral inferior parietal lobule (IPL; left supramarginal gyrus, Brodmann area 40; right angular gyrus, Brodmann area 39), the right middle temporal gyrus (ITG; Brodmann area 38), the right inferior frontal gyrus (IFG; Brodmann area 44), and the right frontopolar cortex (Brodmann area 10).

**Figure 6:**
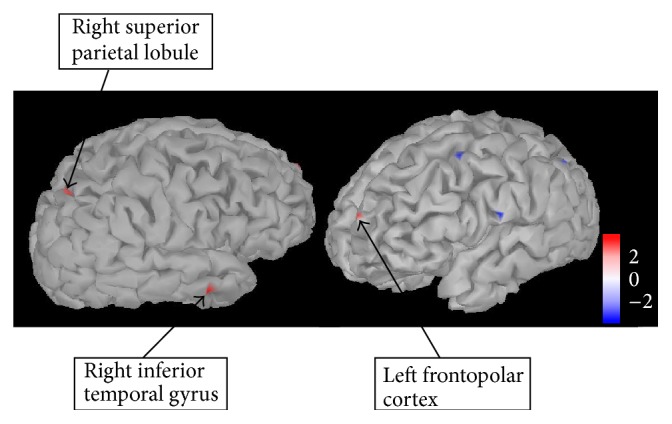
Regions showing significantly higher activation in the AN-R compared to HC group at 320 ms after stimulus. Regions showing significantly higher activation in a *t*-test comparing the AN-R and HC group (shown in red) at 320 ms after stimulus (all food pictures versus baseline). Regions were identified by eye and are uncorrected for multiple comparisons (*p* = 0.005). Scale shown represents the *t*-value. AN-R: recovered Anorexia Nervosa group, HC: healthy control group.

*(2) Source Analysis of Neural Response at 320 ms*. As a significant difference in neural activity at this time point was identified between the AN-R and AN-C group and the AN-R and HC group, differences in source space were investigated in Brainstorm using between-group *t*-tests. Significant regions emerged only when uncorrected for multiple comparisons. As the AN-R group showed increased neural activity compared to the HC and AN-C group, the focus of the source space analysis was also limited to regions of increased activation.  Figure 6 shows regions with increased activation in the AN-R compared to the HC group at 320 ms after stimulus (shown in red, *p* = 0.005). These regions were identified as the right inferior temporal gyrus (Brodmann area 20), the right superior parietal lobule (SPL; Brodmann area 7), and the left frontopolar cortex (Brodmann area 10).

**Figure 7:**
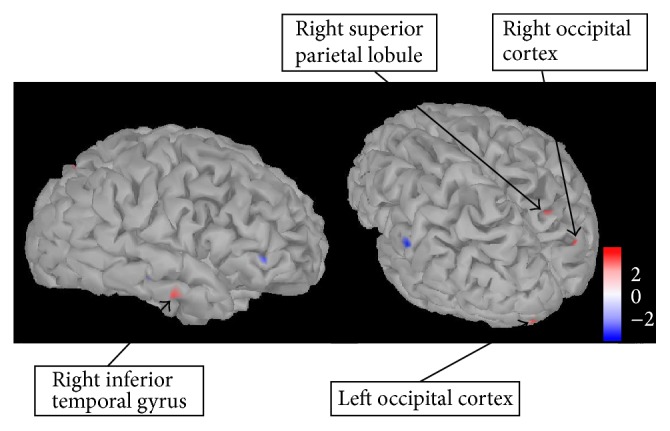
Regions showing significantly higher activation in the AN-R compared to AN-R group at 320 ms after stimulus. Regions showing significantly higher activation in a *t*-test comparing the AN-R and AN-C group (shown in red) at 320 ms after stimulus (all food pictures versus baseline). Regions were identified by eye and are uncorrected for multiple comparisons (*p* = 0.005). Scale shown represents the *t*-value. AN-R: recovered Anorexia Nervosa group, AN-C: current Anorexia Nervosa group.

Figure 7 shows regions with increased activation in the AN-R compared to the AN-C group at 320 ms after stimulus (shown in red, *p* = 0.005). These regions were identified as the right SPL (Brodmann area 7), the left frontopolar cortex (Brodmann area 10), the left occipital cortex (Brodmann area 18), and right occipital cortex (Brodmann area 19).

### 3.4. Correlations with MEG Time-Series Data

As no significant differences were found in the neural response to high- and low-calorie food stimuli, correlations were carried out with the average neural response to* all* food stimuli at each time point of interest. MEG time-series data was averaged over the peak intervals at *p* = 0.01 for the two significant time points (120–180 ms, 300–340 ms).

#### 3.4.1. Correlations with Neural Response at 150 ms

No significant correlation was found with the reaction time data or subjective food picture ratings at this time point in any of the experimental groups. A significant negative correlation was found in the AN-C group with both *Y*-span (*r* = −0.736, *p* = 0.004) and *P*-span (*r* = −0.676, *p* = 0.011). No significant correlation was found with eye-tracking measures in the AN-R and HC groups.

No significant correlation was found with measures of ED symptoms at the time point in any of the experimental groups. A significant correlation was found in the HC group with overall BIS scores (*r* = 0.517, *p* = 0.048) and the BIS nonplanning impulsivity subscale (*r* = 0.625, *p* = 0.013). In the AN-C group, a significant negative correlation was found with the BIS nonplanning impulsivity subscale (*r* = −0.593, *p* = 0.033). No significant correlation was found with any measures of impulsivity or compulsivity in the AN-R group.

#### 3.4.2. Correlations with Neural Response at 320 ms

No significant correlation was found with the reaction time data at this time point in any of the experimental groups. A significant correlation was found in the AN-R group with *P*-span (*r* = 0.599, *p* = 0.031). A significant negative correlation was found in the AN-C group with *Y*-span (*r* = −0.610, *p* = 0.027). A significant correlation was found in the HC group with pleasantness ratings of food stimuli (*r* = 0.590, *p* = 0.021), a correlation not found in the AN groups.

No significant correlation was found with measures of ED symptoms in any of the experimental groups. A significant negative correlation was found in the AN-R group with the BIS nonplanning impulsivity subscale (*r* = −0.584, *p* = 0.028). No significant correlation was found with measures of impulsivity or compulsivity in the AN-C or HC group.

## 4. Discussion

To our knowledge, this is the first study to investigate the neural processing of food stimuli in AN using MEG. Using a time-series analysis, we found a significant main effect of group at two time points of interest in response to both calorie conditions versus baseline: 150 ms and 320 ms after stimulus. Significantly increased neural activity was observed at 150 ms in the AN-C group, compared to both the AN-R and HC groups. In addition, increased neural activity was observed at 320 ms in the AN-R group, in comparison to both the AN-C and HC groups. Despite this enhanced neural activity, both AN groups rated the food pictures as significantly less pleasant than the HC group. Contrary to our hypothesis, we found no significant differences in the neural response to high- versus low-calorie food pictures in any of the groups.

This early increase in neural activity confirms our hypothesis of enhanced early processing of food pictures in AN compared to HCs, which is also seen at a slightly later latency after recovery. These findings are in line with previous behavioral studies indicating an attentional bias for food stimuli in AN [15, 16] and with neuroimaging studies indicating food stimuli are aberrantly salient for individuals with AN [7–10, 13, 49–51].

The absence of nonfood picture stimuli in this study means we cannot rule out the possibility of enhanced neural processing of pictorial stimuli in general in AN. However, our findings show parallels with other electrophysiological studies of food stimuli processing in AN, which have also indicated enhanced early processing of food pictures using EEG [22, 23]. Blechert et al. [23] reported enhanced neural activity at 200–300 ms (the Early Positive Negativity; EPN) after stimulus to both high- and low-calorie food pictures in acute AN, compared to enhanced activity for high-calorie foods only observed in HCs, suggesting a generalized bias towards food stimuli in AN [23]. Furthermore, Novosel et al. [22] reported an increased P300 response to food stimuli, but not to emotional or neutral stimuli in individuals with AN compared to HCs [22]. These results are consistent with the motivated guidance of attention towards disorder-related stimuli in AN, which may allow preferential processing of these stimuli at a later stage [23]. However, both of these studies also reported a significant difference in the response to high- and low-calorie pictures in AN, a differentiation which was not observed in the current study. This may be due to methodological differences between these studies and the current paradigm, such as the inclusion of neutral or nonfood stimuli or insufficient statistical power in picking up subtle differences, due to small sample size.

The different latencies of increased neural activity in the AN-C and AN-R groups may reflect enhanced processing at different stages in the information processing stream. The earlier increase in neural activity found in the AN-C group is at a latency associated with early sensory processing and attentional orienting/selection processes [18]. An increase in activity at 100 ms after stimulus has been shown in response to fearful and threatening stimuli in those with high trait anxiety [52, 53]. As such, these results may reflect an increased threat response, or increased vigilance, very early in the neural processing of food stimuli in individuals with AN. This supports suggestion that individuals with AN show hypersensitivity towards food stimuli, which may enable subsequent avoidance of these stimuli and maintenance of dietary restraint [13]. The later latency of enhanced neural activity in the AN-R is consistent with increased emotional processing of stimuli [19, 20]. Indeed, an increase in activity around 300 ms has been reported in response to emotional pictures (appetitive or aversive) [54, 55], suggesting food stimuli are experienced as emotionally significant even after recovery from AN. It could be hypothesized that recovery from AN requires that food is no longer avoided as in the acute state, but it may still hold increased emotional salience for these individuals, resulting in increased emotional processing at this latency.

The results of the time-series analysis are further supported by an analysis of group differences in source space. It should be emphasized that group differences in source space were only observed when uncorrected for multiple comparisons, which is likely due to low statistical power. However, the regions identified are consistent with both hypersensitivity and increased cognitive control in response to food pictures, previously suggested in neuroimaging and electrophysiological studies in AN [13, 22, 23, 50, 51]. Increased neural activity at 150 ms after stimulus in the AN-C group was observed in the primary motor cortex (precentral gyrus), previously suggested to reflect an increased appetitive response to food pictures in AN [50]. Furthermore, increased activity in the AN-R group at 320 ms was observed in the SPL and ITG, suggesting increased visual and spatial processing of food stimuli even after recovery [56, 57].

Increased activation of the frontal pole, responsible for complex decision-making and cognitive control [58], was observed in both the AN-C group at 150 ms and the AN-R group at 320 ms, compared to the HC group. This finding mirrors that of our recent fMRI study using a parallel food pictures paradigm, in which we found differential activation of the frontal pole to high-calorie foods in AN; the opposite of that was found in controls (unpublished data). Activation of the frontal pole in both acute and recovered AN may represent trait-related activation of regions associated with higher-order cognitive processing in response to food. This is supported by a significant negative correlation in both AN groups, between neural activity at 150 ms, and the BIS nonplanning impulsivity subscale. This suggests that, in both AN groups, higher neural activity at 150 ms after stimulus was associated with a tendency to plan ahead and to think about future consequences. Whilst speculative, this may reflect the activation of higher-order cognitive control processes to downregulate increased attention for food stimuli and enable subsequent avoidance of food in AN.

The different latencies of increased neural activity between the AN-C and AN-R groups were also reflected by group differences in source space. At 150 ms, the AN-C group showed increased activation in regions associated with attention and response inhibition (IPL, frontal pole, and IFG) [59–61], although again this was only observed when uncorrected for multiple comparisons. Activation of these regions again suggests a possible state-related enhancement of both attention and response inhibition towards food stimuli in acute AN compared to recovery. Furthermore, at 320 ms, the AN-R group showed increased activation in regions consistent with increased visual processing of food stimuli [56, 57]. This is further supported by eye-tracking data, indicating significantly increased exploration of food stimuli in the AN-R group, compared to the AN-C and HC groups. Furthermore, neural activity at 320 ms was positively correlated with the *P*-span eye-tracking measure in the AN-R group. These results raise the possibility that this increase in neural activity in the AN-R group represents an increase in the visual P300 component. Modulation of this component is related to motivation/emotional properties of visual stimuli [21], and it has been associated with overfocused attention and hyperaroused cognitive processes [62]. As such, it is possible that food stimuli are associated with an increase in emotional and cognitive processing in recovery, in contrast with the AN-C group, for which increased activity at 150 ms was negatively correlated with measures of eye tracking. Whilst speculative, it could be hypothesized that increased cognitive/emotional processing of food stimuli following recovery represents a reversal of avoidance of food seen in the acute state of AN.

### 4.1. Limitations

Some methodological limitations are of note. As with many studies in this area, the sample size used was fairly small; however, we did isolate a sample of purely restrictive AN. Many neuroimaging studies in AN have included both restrictive and binge/purge AN subtypes and as such are unable to control for the possibility of within-group variability in neural activity, which may be related to important differences in psychopathology between subtypes of AN. However, future research may benefit from larger sample sizes enabling the inclusion and comparison of both AN subtypes. In addition, a number of participants had comorbid anxiety or depression diagnoses and were taking serotonergic antidepressants and/or antipsychotic medications. Antidepressant and antipsychotic medications have been shown to alter neural activity measured by MEG [63, 64], and so the effects of these medications, or indeed comorbid diagnoses, on our results cannot be ruled out.

An important limitation of the experimental paradigm was that it did not include nonfood pictures as a control condition, and as such we cannot rule out the possibility that individuals with AN would show an increased neural response to viewing pictures in general. In addition, significant differences in source space were only observed when uncorrected for multiple comparisons, which is likely due to low statistical power. As such, these results should be interpreted with caution, and replication is needed with a larger sample size and the inclusion of nonfood stimuli for comparison. However, previous studies corroborate our findings in source space [13, 22, 23, 50, 51] and show individuals with AN do not differ from healthy individuals in neural response to nonfood images [22, 23].

### 4.2. Conclusions

In conclusion, we observed early enhanced activity for both high- and low-calorie foods in individuals with acute AN, compared to HCs. This finding is consistent with an early attentional bias for food stimuli in AN, which appears to occur at a later latency in recovery. Whilst uncorrected for multiple comparisons, source space analysis indicated increased activity in regions associated with attention and cognitive control in the AN groups. This is consistent with previous neuroimaging and electrophysiological studies and may enable identification and subsequent avoidance of salient food stimuli and the maintenance of dietary restraint in AN. The later latency of increased activity in the AN-R group may reflect a reversal of this avoidance, with increased visual and cognitive processing of food stimuli in this group.

## Supplementary Material

The supplementary materials provide extra information and details regarding the methods and results of the study. Examples of the high- and low-calorie food pictures used in the task are provided. For further details of behavioral results, groups means and differences for VAS scale ratings, food picture ratings, reactions times and eye tracking measures are also provided.

## Figures and Tables

**Table 1:** Demographic and psychological characteristics stratified by group.

Variable	AN-C (*n* = 13)		AN-R (*n* = 14)		HC (*n* = 15)		Significance (*p* value)	Post hoc (*p* < 0.05)
	Mean	SD	Mean	SD	Mean	SD		
Age	31.2	5.3	27.1	6.5	23.7	5.4	0.018	AN-C > HC
BMI	15.7	1.9	20.9	1.6	21.4	1.9	<0.001	AN-C < AN-R, HC
NART	114.0	7.5	114.6	6.8	109.1	7.3	0.087	—
Lowest BMI	13.0	1.2	13.8	1.2	—	—	0.571	—
Duration of illness	11.0	5.7	5.7	4.2	—	—	0.252	—
Age of onset	21.7	6.1	16.5	2.1	—	—	<0.001	AN-C > AN-R
EDE	3.1	1.4	0.74	0.6	0.24	0.2	<0.001	AN-C > AN-R, HC
EDE-Q	3.4	1.7	0.95	0.8	0.50	0.3	<0.001	AN-C > AN-R, HC
SS	52.8	28.7	5.3	8.4	0.27	0.8	<0.001	AN-C > AN-R, HC
BIS	56.5	12.5	61.6	9.7	56.0	11.8	0.363	—
CIA	29.5	13.4	7.9	6.5	1.9	2.3	<0.001	AN-C > AN-R, HC
OCI-r	20.8	15.8	10.8	6.4	5.7	6.8	0.002	AN-C > AN-R, HC
BDI	29.6	18.1	6.4	6.4	3.4	4.6	<0.001	AN-C > AN-R, HC
SPSRQ Punishment	15.5	5.9	12.1	4.7	8.4	4.0	0.001	AN-C > HC
SPSRQ Reward	8.1	4.3	8.9	4.3	9.3	3.4	0.707	—
STAI state	50.1	13.0	34.4	5.4	26.5	6.5	<0.01	AN-C > AN-R > HC
STAI trait	60.7	12.9	45.2	10.6	32.00	9.4	<0.001	AN-C > AN-R > HC
YBC-EDS-SRQ, current	16.4	8.2	3.8	3.9	0	0	<0.001	AN-C > AN-R, HC
YBC-EDS-SRQ, past	24.6	5.3	23.8	5.3	0	0	<0.001	AN-C, AN-R > HC

BMI: body mass index; NART: National Adult Reading Test; EDE: Eating Disorder Examination; EDE-Q: Eating Disorder Examination-Questionnaire; SS: Self-Starvation Scale; BIS: Barratt Impulsiveness Scale; CIA: Clinical Impairment Assessment; OCI-r: Obsessive Compulsive Inventory Revised; BDI: Beck Depression Inventory; YBC-EDS-SRQ: Yale-Brown-Cornell Eating Disorder Scale Self-Report Questionnaire; STAI: State Trait Anxiety Inventory; — indicates no significant difference between groups.
