# Supplementary material for: Enhanced Early Neuronal Processing of Food Pictures in Anorexia Nervosa: A Magnetoencephalography Study
Source: Psychiatry J. 2016 Jul 25;2016:1795901. doi: 10.1155/2016/1795901 (PMC4976260; doi:10.1155/2016/1795901)
Supplement: Supplementary file 1 — The supplementary materials provide extra information and details regarding the methods and results of the study. Examples of the high- and low-calorie food pictures used in the task are provided. For further details of behavioral results, groups means and differences for VAS scale ratings, food picture ratings, reactions times and eye tracking measures are also provided. [file 1795901.f1.docx]

**Supplementary Materials**

**
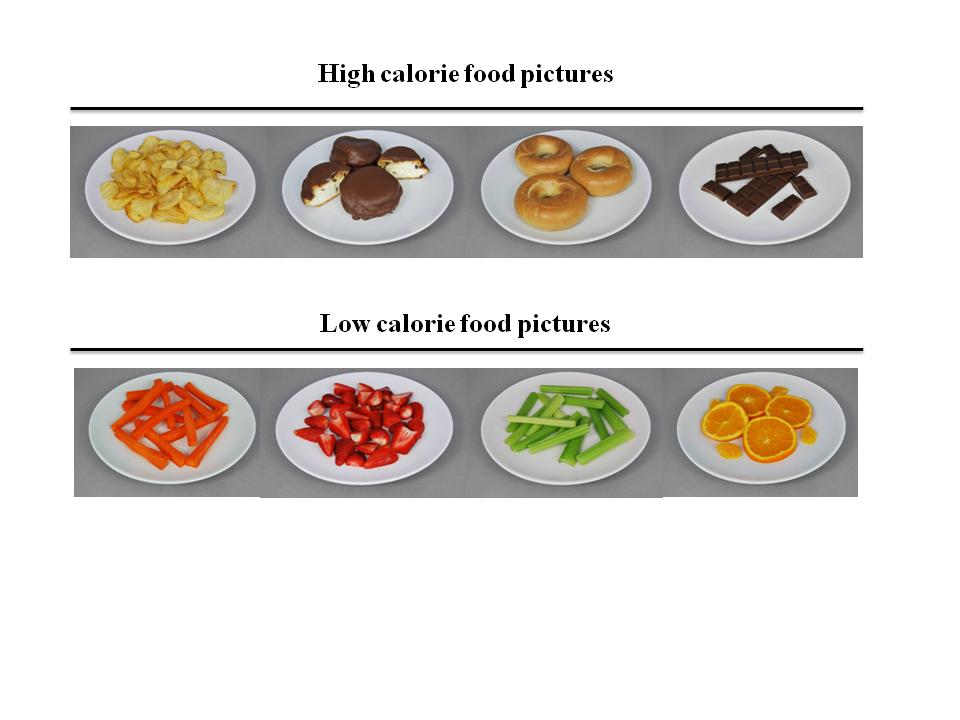
Figure S1. Examples of high and low calorie pictures presented in the Food Pictures Task.**

**Table S1. VAS scale ratings stratified by group**.

| **VAS scale** | **AN-C (n=13)** | | **AN-R (n=14)** | | **HC (n=15)** | | **Significance (p value)** | **Post hoc (p<.05)** |
| --- | --- | --- | --- | --- | --- | --- | --- | --- |
| I feel…. | **Mean** | **SD** | **Mean** | **SD** | **Mean** | **SD** |  |  |
| **Happy** | 48.8 | 27.2 | 57.2 | 26.2 | 72.8 | 17.6 | **0.034** | HC>AN-C |
| **Despondent** | 30.9 | 26.7 | 27.4 | 22.2 | 20.5 | 21.0 | 0.484 | --- |
| **Anxious** | 32.9 | 26.4 | 22.9 | 20.2 | 21.1 | 24.6 | 0.389 | --- |
| **Hungry** | 47.3 | 26.5 | 61.1 | 26.3 | 78.9 | 16.4 | **0.004** | HC>AN-C |
| **Thirsty** | 45.8 | 32.0 | 53.7 | 25.1 | 65.9 | 22.0 | 0.140 | --- |
| **Full** | 34.6 | 28.5 | 22.1 | 29.0 | 13.7 | 12.3 | 0.086 | --- |
| **Desire to eat** | 47.0 | 31.7 | 65.6 | 26.5 | 80.5 | 12.9 | **0.004** | HC>AN-C |
| **Fat** | 52.8 | 27.4 | 56.4 | 13.6 | 45.8 | 15.6 | 0.337 | --- |
| **Afraid of weight gain** | 80.2 | 19.4 | 55.9 | 25.3 | 33.6 | 19.9 | **<0.001** | AN-C>AN-R>HC |

# Table S2. Subjective food picture ratings, reaction times, and eye-tracking measures stratified by experimental group and calorie.

| **Variable** | **AN-C (n=13)** | | **AN-R (n=14)** | | **HC (n=15)** | |
| --- | --- | --- | --- | --- | --- | --- |
|  | **High (SD)** | **Low (SD)** | **High (SD)** | **Low (SD)** | **High (SD)** | **Low (SD)** |
| **Subjective Pleasantness (out of 10)** | 4.26 (1.62) | 2.87 (1.59) | 4.81 (1.86) | 3.92 (1.69) | 6.53 (1.66) | 6.28 (2.01) |
| **Subjective Wanting (out of 10)** | 5.13 (2.05) | 4.37 (1.99) | 5.18 (1.34) | 4.51 (1.52) | 5.64 (1.62) | 5.56 (1.60) |
| **Reaction Time (ms)** | 507.8 (112.3) | 473.6 (110.5) | 465.4 (115.9) | 423.0 (46.1) | 439.0 (68.1) | 423.4 (45.3) |
| **X-Span (mm)** | 1.28 (0.92) | 1.11 (0.82) | 1.50 (1.10) | 1.32 (1.06) | 1.36 (1.13) | 1.12 (0.82) |
| **Y-Span (mm)** | 1.13 (0.82) | 1.03 (0.73) | 1.35 (1.01) | 1.19 (0.96) | 1.12 (1.06) | 0.94 (0.82) |
| **P-Span (mm)** | 0.44 (0.34) | 0.40 (0.28) | 0.54 (0.28) | 0.28 (0.29) | 0.44 (0.40) | 0.41 (0.21) |
